# Development of an Early Alert System for an Additional Wave of COVID-19 Cases Using a Recurrent Neural Network with Long Short-Term Memory

**DOI:** 10.3390/ijerph18147376

**Published:** 2021-07-09

**Authors:** Finn Stevenson, Kentaro Hayasi, Nicola Luigi Bragazzi, Jude Dzevela Kong, Ali Asgary, Benjamin Lieberman, Xifeng Ruan, Thuso Mathaha, Salah-Eddine Dahbi, Joshua Choma, Mary Kawonga, Mduduzi Mbada, Nidhi Tripathi, James Orbinski, Bruce Mellado, Jianhong Wu

**Affiliations:** 1 School of Physics, Institute for Collider Particle Physics, University of the Witwatersrand, Johannesburg 2050, South Africa; finn.david.stevenson@cern.ch (F.S.); 716034@students.wits.ac.za (B.L.); Xifeng.Ruan@wits.ac.za (X.R.); 1144845@students.wits.ac.za (T.M.); salah-eddine.dahbi@cern.ch (S.-E.D.); nalamotse.joshua.choma@cern.ch (J.C.); nidhi.tripathi@cern.ch (N.T.); bruce.mellado@wits.ac.za (B.M.); 2 School of Computer Science and Applied Mathematics, University of the Witwatersrand, Johannesburg 2050, South Africa; Kentaro.Hayashi@students.wits.ac.za; 3 Laboratory for Industrial and Applied Mathematics, Department of Mathematics and Statistics, York University, Toronto, ON M3J 1P3, Canada; jdkong@yorku.ca (J.D.K.); wujhhida@gmail.com (J.W.); 4 Disaster & Emergency Management, School of Administrative Studies and Advanced Disaster, Emergency and Rapid-Response Simulation, York University, Toronto, ON M3J 1P3, Canada; asgary@yorku.ca; 5 Department of Community Health, School of Public Health, University of the Witwatersrand, Johannesburg 2050, South Africa; Mary.Kawonga@wits.ac.za; 6 Office of the Premier, Gauteng Government, 13th Floor, East Wing, 30 Simmonds St., Marshalltown, Johannesburg 2107, South Africa; Mduduzi.Mbada@gauteng.gov.za; 7 Dahdaleh Institute for Global Health Research, York University, Toronto, ON M3J 1P3, Canada; orbinski@yorku.ca; 8 iThemba LABS, National Research Foundation, P.O. Box 722, Somerset West 7129, South Africa

**Keywords:** COVID-19, South Africa, early detection, crisis management, daily case prediction, Recurrent Neural Network with Long Short-Term Memory

## Abstract

The impact of the still ongoing “Coronavirus Disease 2019” (COVID-19) pandemic has been and is still vast, affecting not only global human health and stretching healthcare facilities, but also profoundly disrupting societal and economic systems worldwide. The nature of the way the virus spreads causes cases to come in further recurring waves. This is due a complex array of biological, societal and environmental factors, including the novel nature of the emerging pathogen. Other parameters explaining the epidemic trend consisting of recurring waves are logistic–organizational challenges in the implementation of the vaccine roll-out, scarcity of doses and human resources, seasonality, meteorological drivers, and community heterogeneity, as well as cycles of strengthening and easing/lifting of the mitigation interventions. Therefore, it is crucial to be able to have an early alert system to identify when another wave of cases is about to occur. The availability of a variety of newly developed indicators allows for the exploration of multi-feature prediction models for case data. Ten indicators were selected as features for our prediction model. The model chosen is a Recurrent Neural Network with Long Short-Term Memory. This paper documents the development of an early alert/detection system that functions by predicting future daily confirmed cases based on a series of features that include mobility and stringency indices, and epidemiological parameters. The model is trained on the intermittent period in between the first and the second wave, in all of the South African provinces.

## 1. Introduction

The first “Coronavirus Disease 2019” (COVID-19) cases were discovered from an initial cluster of pneumonia of unknown etiology in the metropolitan city of Wuhan, province of Hubei, mainland China, in late December 2019 [1]. It is caused by an infectious agent known as “Severe Acute Respiratory Syndrome-related Coronavirus type 2” (SARS-CoV-2), the contraction of which results in a generally mild or even asymptomatic infection, that can, in a fraction of patients, evolve into a serious, life-threatening communicable disease [2]. The impact of the still ongoing pandemic has been and is still vast, affecting not only global human health and stretching healthcare facilities, but also profoundly disrupting societal and economic systems worldwide [3].

The nature of the way the virus spreads causes cases to come in further recurring waves. This is due a complex array of biological, societal and environmental factors, including the novel nature of the emerging pathogen, for which there was no community cross-protective immunity, with the population being substantially naive to the virus [4]. Thanks to unprecedented global efforts and co-operations, several candidate vaccines have been developed, tested and some of them have been finally approved [5]. However, despite excellent efficacy and safety profiles, there is still uncertainty about the length of the protection conferred by approved vaccines [6] and, moreover, the immunization campaigns in different countries are still lagging behind, facing organizational difficulties and scarcity of doses as well as of human resources [7]. Other determinants of the epidemic trends of the COVID-19 pandemic include seasonal factors [8], and meteorological drivers [9], as well as community heterogeneity and complex, highly heterogeneous social networks, with phenomena such as over-dispersion, super-spreading events, super-spreaders [10] and behavioral changes at the population level [11]. All these variables, and especially the behavioral ones [12], make the COVID-19 transmission dynamics particularly uneven and recurring, by challenging the full attainment of the herd immunity, with only a transient, waning collective immunity being achieved [12].

Further, the public health measures implemented and enforced by the country authorities, known as Non-pharmaceutical Interventions (NPIs), are not sustainable and acceptable by the populations for long periods, resulting into “cyclical lock-downs” [13] based on data-driven escalating/de-escalating, shutting down/re-opening strategies. These cycles of strengthening and easing/lifting of the mitigation interventions are among the factors contributing to the recurring nature of the ongoing COVID-19 pandemic [13].

Given such a cyclical nature of the COVID-19 outbreak, it is, therefore, crucial to have an early alert system to identify when another wave of cases is about to occur, especially considering that COVID-19 could become a recurrent seasonal infection [14]. The availability of a variety of newly developed indicators allows for the exploration of multi-feature prediction models for COVID-19 case data. Ten indicators were selected as features for our prediction model. The model chosen is a Recurrent Neural Network (RNN) with Long Short-Term Memory (LSTM). RNNs with LSTM are known to be good time-series predictive models, especially for multi-feature model architectures that require a memory component without the vanishing gradient pitfalls of a normal RNN [15].

This paper documents the development of an early alert/detection system that functions by predicting future daily confirmed cases based on a series of features that include mobility and stringency indices, and epidemiological parameters, exploiting Big Data and Artificial Intelligence. The model is trained on the intermittent period in between the first and the second wave, in all of the South African provinces. The COVID-19 case prediction parameter chosen was the daily change in cases, dTCt. The chosen model was trained on data in the interim period between two COVID-19 case peaks. This caused the system to be able to predict daily cases accurately during the interim period; however, when there is a COVID-19 case peak, the system is unable to recognise the behaviour of the features in relation to the prediction parameter dTCt. We have taken advantage of the pitfall of the model to predict the daily cases as soon as a peak is reached, in order to develop the early detection system. A warning was created to notify the government and general public when the relative difference formula of the actual versus predicted daily cases exceeds the province specific threshold value for the relative difference, computed as Risk Index Metric (RIM).

Using real-world data from in between the first and second waves to calibrate the model and using the second peaks data as verification of the correct functioning of the model, the system was able to accurately identify and confirm the beginning of the second wave. All provinces in South Africa were used to verify that the earlier detection system functions to identify the beginning of the second wave. The model is now being used for surveillance of the third wave in South Africa.

## 2. Materials and Methods

### 2.1. Description of Features (Data)

The following section will provide a brief overview of the various indicators that were used as features for the RNN with LSTM model.

#### 2.1.1. Mobility Indicators

Since the beginning of the COVID-19 pandemic, Google and Facebook have produced mobility reports that include different types of mobility indicators as a measure to understand the consequence of implemented regulations and NPIs on the public movement and social interactions. These indicators can be used as valuable inputs to the model. Each of the mobility reports includes different types of mobility indicators that are developed using different methodologies.

Table 1 contains details on all the mobility indicators used in our model as features:

The Google Mobility Report data is useful for understanding the geo-spatial movement of people during the pandemic [16]. Movement trends of people over time and over different categories of places are tracked. The report contains three location categories. The categories are titled: ‘retail & recreation’; ‘groceries & pharmacies’; ‘parks’, ‘transit stations’; ‘workplaces’ and ‘residential’. These indicators are a valuable resource for understanding how people interact with different types of locations. All of the Google mobility indicators have the same overall trend with minor difference except the residential which has an almost opposite behaviour due to the increase of people staying in their homes as a result of the pandemic.

The Facebook movement data sets were developed to assist researchers and public health experts in monitoring and tracking how populations are responding to social/physical distancing measures [17]. The Facebook mobility report contains two complementary indicators to describe changes in movement over time: namely, ‘Change in Movement’ and ‘Stay Put’. Each of the indicators provides different perspectives on movement trends. The Facebook mobility report methodology divides geographical areas up into equal area tiles. The ’Change in Movement’ indicator measures the number of tiles people are visiting in a day in a specific region with respect to a baseline defined as the average number of tiles visited daily in the month of February 2020. The ‘Stay Put’ indicator conversely measures how many people are staying within a single tile area for the whole day compared to the February baseline. People who use Facebook on a mobile device have the choice of providing their precise location. Movement Range Trends are produced by aggregating this data.

#### 2.1.2. Stringency Indicators

Another valuable type of indicator to be considered as a feature for the model is a policy stringency indicator. There are a number of stringency indicators that have been developed as indications of the level or strictness of implemented regulation in a specific country or region. Arguably the most comprehensive stringency indicator that has been developed is the Oxford COVID-19 Government Response Tracker (OxCGRT stringency index) [18]. The OxCGRT Stringency index is made up of a number of NPI containment and closure policy indicators which are scored, summed up and then averaged to achieve the final stringency value for any given day. Details on each of the chosen containment indicators and their coding can be seen in the OxCGRT code book.

#### 2.1.3. Epidemiological Parameters

The specific epidemiological parameter used as the prediction parameter in this research is the number of new daily COVID-19 cases.

### 2.2. Data Preprocessing

The data preprocessing required for the system can be divided into two separate sections: the primary and secondary data preprocessing. The primary data preprocessing consists of the conversion of the multiple different data sources (COVID-19 case data, Facebook mobility, Google mobility and OxCGRT) from their stock format (long format) into time-series format so that each variable that will be used as a feature exists in its own column in a final time-series data-frame. The secondary data preprocessing involves feature scaling and finally the re-framing of the multivariate time series into a supervised learning data-set that incorporates the selected window size chosen. The supervised learning data format created contains new columns that represent the variables from previous time steps. The value of the chosen window size determines how many new columns will be created for each specific feature. For example, if the window size is three, three new columns will be created for each variable. The first new column will contain the data from the original column shifted one time-step down, and the second added column will contain the original data shifted twice and so on. This is to incorporate the ability of the LSTM RNN to observe previous values of features when predicting the new value of a chosen feature.

### 2.3. Research Methodology

The aim of the present research is to develop a functional alert system for an additional wave of COVID-19 cases in a specific region. The regions used for the research are all the provinces of South Africa. The approach is to do time-series prediction of a chosen epidemiological parameter based on a collection of mobility, stringency parameters and epidemiological parameters.

This research used a confirmatory approach, where the objective was to find out if the idea was supported by the data. The data from the second wave of COVID-19 cases in South Africa was used to verify the model.

**Figure 1:**
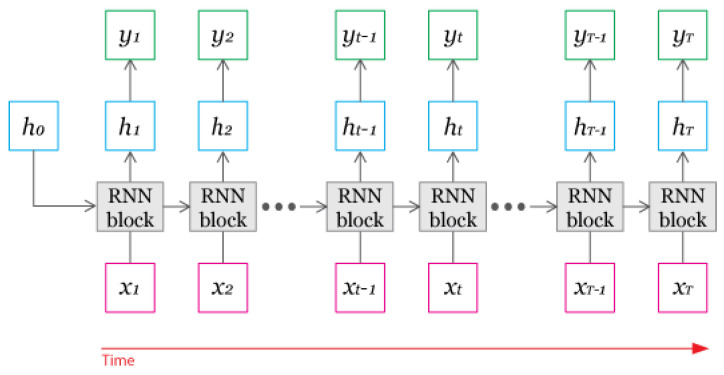
Recurrent Neural Network (RNN) structure.

**Figure 2:**
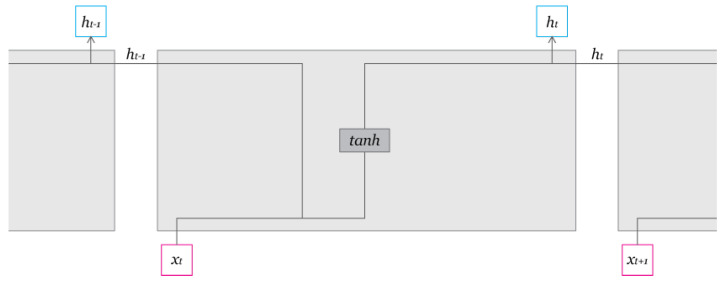
Details of the Simple Recurrent Neural Network (RNN) Block.

**Figure 3:**
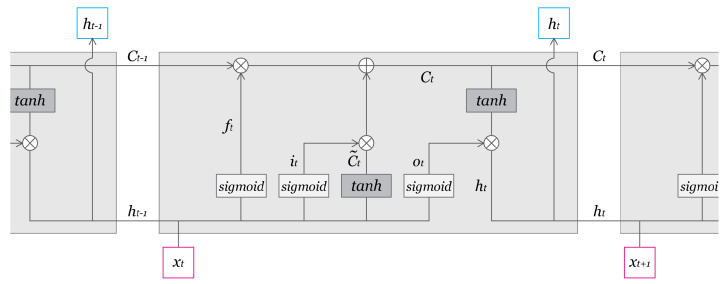
Details of the Long Short-Term Memory (LSTM) Recurrent Neural Network (RNN) Block.

The chosen prediction model is a RNN with LSTM. This model architecture allows for multi-feature and multi-step predictions. Though standard RNNs are often used in time series prediction, the standard architecture suffers from the problem of vanishing gradients, which hinders the learning of long period relationships and patterns in data sequences. RNNs with LSTM solve the issue by deliberately adding long-term memory [19,20]. RNNs with LSTM have two memory cells, one for long-term memory and another for short-term memory to solve the problem. The equations below describe the LSTM RNN block. $C_{t}$ is responsible for long-term memory and $h_{t}$ for short-term memory. The introduction of the Forget gate vector, proposed by Felix A. Gers et al. in 1999, has also improved the accuracy of the prediction by allowing the adjustment of long-term memory [21]. More in detail, the Forget gate vector, $f_{t}$, controls how much information is discarded from long-term memory, $C_{t-1}$. The new long-term memory cell, $C_{t}$, is created by adding information from the input gate vector, $i_{t}$, and the new short-term memory cell, $h_{t}$ is decided by the output vector, $o_{t}$ and the long-term memory, $C_{t}$ (Figure 1, Figure 2 and Figure 3).

(1) $$\begin{array}{ccc} f_{t} & = & \sigma (W_{f}\cdot \left[h_{t-1},x_{t}\right]+b_{f}) \end{array}$$

(2) $$\begin{array}{ccc} i_{t} & = & \sigma (W_{i}\cdot \left[h_{t-1},x_{t}\right]+b_{i}) \end{array}$$

(3) $$\begin{array}{ccc} \tilde{C_{t}} & = & tanh(W_{c}\cdot \left[h_{t-1},x_{t}\right]+b_{c}) \end{array}$$

(4) $$\begin{array}{ccc} C_{t} & = & f_{t}⊙C_{t-1}+i_{t}⊙\tilde{C_{t}} \end{array}$$

(5) $$\begin{array}{ccc} o_{t} & = & \sigma (W_{o}\cdot \left[h_{t-1},x_{t}\right]+b_{0}) \end{array}$$

(6) $$\begin{array}{ccc} h_{t} & = & o_{t}⊙tanh\left(C_{t}\right) \end{array}$$

where ⊙ represents the Hadamard product.

The main constraints related to the formation of an early detection algorithm are related to the availability of mobility and epidemiological data. The Google and Facebook mobility reports are available every Sunday with week-old data. This must be taken into consideration when developing the functioning of the alert system.

**Figure 4:**
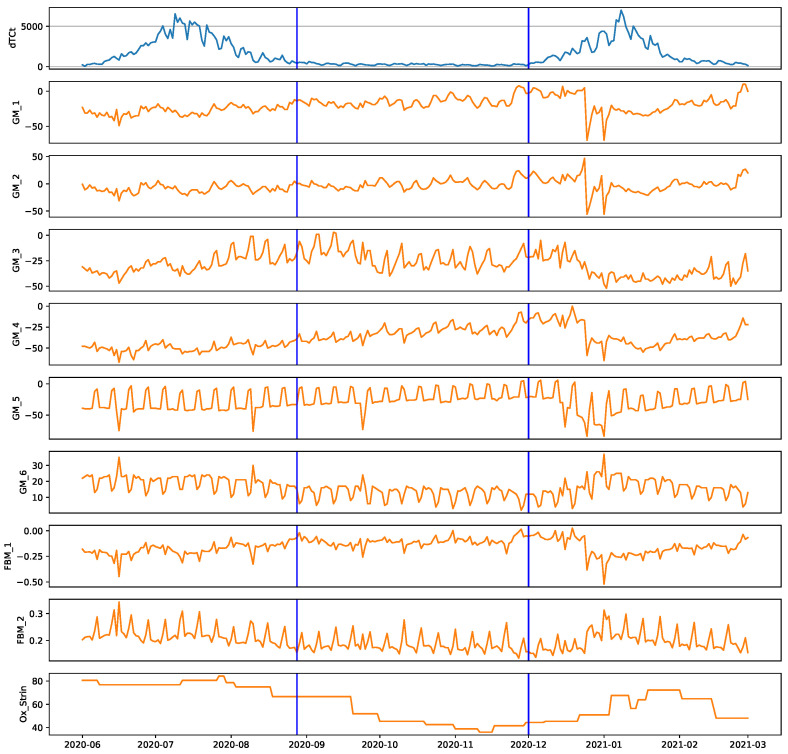
Appropriate test-train period for Gauteng Province, South Africa.

The model was trained on data from the interim period between the first and second COVID-19 case waves experienced in South Africa. This provided the model with the ability to predict daily cases accurately during the interim period; however, when there is a COVID-19 case peak, the system is unable to recognise the behaviour of the features in relation to the prediction parameter dTCt. The pitfall of the model to predict the daily cases during a peak, has been taken advantage of to develop the early detection system. Figure 4 shows the total period applicable for training of the model in between peak one and peak two for the Gauteng province.

A schematic of the neural network architecture can be found in Appendix A.

#### 2.3.1. Model Outputs

The output of the trained RNN model with LSTM is a 14-day prediction of new daily cases, dTCt. The first date of the 14-day prediction is a Monday. This Monday corresponds to 6 days earlier than the actual date that the model is run, this is due to the external constraints of data availability from each source. This means that the prediction will run only 7 days into the future from the date the prediction is done.

A secondary output of the model that can be obtained daily is the relative difference between the prediction and the actual recorded value. The formula for the relative difference is:

(7) $$R_{D}=\frac{\left(dTCt_{A}-dTCt_{P}\right)}{dTCt_{P}}$$

where $dTCt_{A}$ = Daily change in actual total cases and $dTCt_{P}$ = Daily change in predicted total cases.

The relative difference is the chosen RIM for an alert of an additional COVID-19 case wave.

In accordance with data availability, the re-calibration of the model and the 14-day dTCt prediction is done every Sunday, producing prediction values for the dates from the Monday before to the Monday after. It is important to note that when the alert system functions as a surveillance system for the third wave, the second peak is removed from the training data of the model.

#### 2.3.2. Hyper-Parameter Optimization

In order to further refine the functioning of the model to identify an additional case wave, the hyper-parameters of the LSTM RNN were optimised using a manual optimisation method consisting of nested loops that looped through a range of possible values for four chosen model hyper-parameters, whilst recording an evaluation metric for each of the combination of hyper-parameters. The value options of the hyper-parameter are shown in Table 2. The evaluation metric used for this optimization was made by summing the absolute value of each $R_{d}$ value created from three different non-overlapping 14 day prediction periods. Three different 14 non-overlapping prediction periods were used for the optimisation for cross validation purposes and to reduce the possibility of over-fitting based on optimising using only one 14-day prediction period.

#### 2.3.3. Methodology Comparison

**Figure 5:**
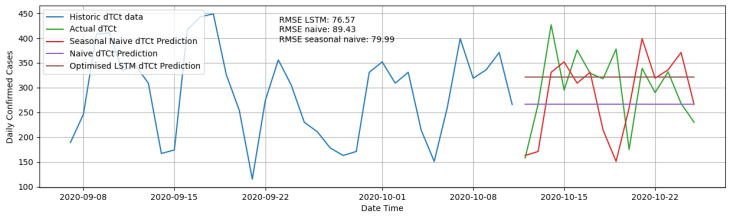
Comparison of LSTM RNN prediction to naive forecast and seasonal naive forecast.

For the purpose of validation of this methodology, the prediction of dTCt and associated RMSE using the LSTM RNN model were compared to alternative more trivial methodologies. The first methodology chosen for the comparison is a naive forecast. A naive forecasting the context of a 14-day prediction is created by projecting the last actual dTCt value available forward for 14 days. Taking the naive forecast slightly further, the second methodology used for the comparison is a seasonal naive forecast. Which in this case creates a 14-day prediction that is equal to not just the last actual value, but the last 14 actual values. Usually, seasonal naive forecasts are performed using monthly, quarterly or yearly seasonality, but in the context of predicting dTCt, weekly seasonality is sometimes evident due to case data reporting patterns. These two more trivial methodologies for predicting dTCt are compared to the RNN LSTM methodology by choosing a prediction date in an interim period and comparing the RMSE over the prediction period. The RMSE results can be seen in Table 3 and a comparison of their predictions is shown in Figure 5.

It can be seen that the RMSE for the LSTM RNN methodology proved to be lower for the chosen comparison date. Although the seasonal naive forecast performed relatively well over the chosen prediction period, this is not always the case due to the fact that reporting patterns change over time due to inconsistencies in human-centric reporting systems. Therefore, we conclude that the more informed straight line prediction produced by the LSTM RNN model proved to be best.

#### 2.3.4. Flow Diagram of System

**Figure 6:**
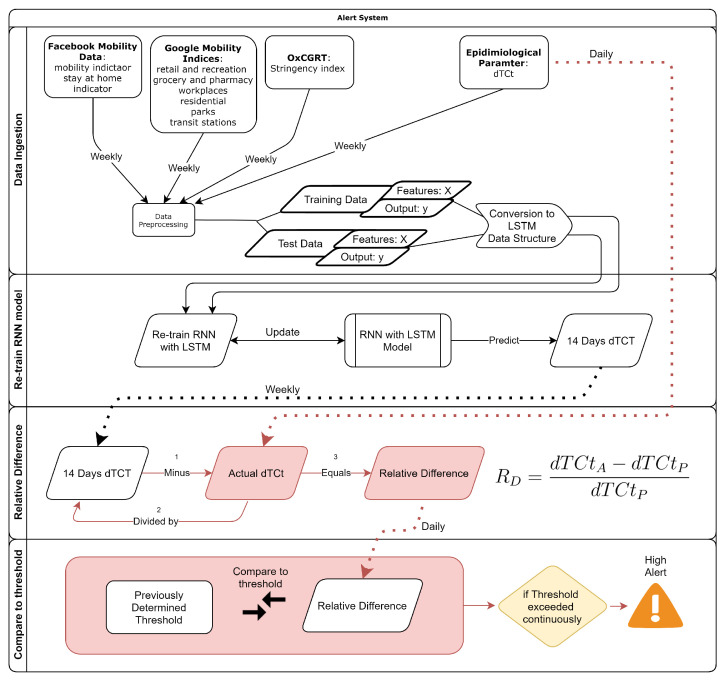
Flow Diagram of the developed Alert System related to COVID-19.

The following diagram (Figure 6) provides a graphical representation of the working of the alert system. Data channels and processes marked in red happen on a daily basis, whilst black data channels and all other processes happen weekly. The model is re-calibrated weekly and predictions are made weekly; however, relative difference values are obtained daily when the actual case data becomes available.

The first block labeled ’Data Ingestion’ in Figure 6 above represents both the data ingestion from the various sources, and the primary and secondary data prepossessing steps. The output of the first block is a supervised time-series format dataset that is appropriate for an RNN with LSTM for a chosen window size. The ’Re-train RNN model’ block demonstrates how the model is re-trained weekly and the output of this block is a 14 day prediction of dTCt. The ’Relative Difference’ block shows that the relative difference value (the RIM) is calculated daily when new case data becomes available. Lastly, the ’Compare to threshold’ block demonstrates how the RIM is compared to a previously obtained threshold in order to determine when and if the threshold is exceeded continuously, which would signify another case wave commencing.

### 2.4. Province Specific Risk Index Threshold

**Figure 7:**
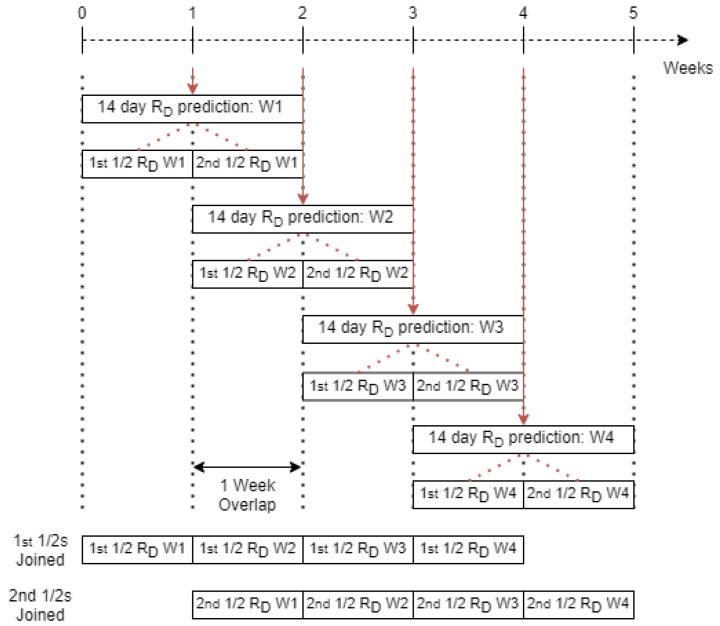
Diagram showing overlap of *R_D_* values obtained from each weeks prediction.

For each province in South Africa, a threshold for the RIM is found by comparing the distribution of the $R_{D}$ values over non-peak periods and peak periods. It is evident that the distribution of $R_{D}$ over a peak period is much highly skewed than that of non-peak periods. The $R_{D}$ distributions are analysed by separating the $R_{D}$ values obtained over peak and non-peak times into all the values from the first half for the 14-day prediction and from the last half of the 14-day prediction. This separation is done in order to account for the overlap of $R_{D}$ values caused by doing a 14-day prediction every 7 days. Figure 7 visualises the $R_{D}$ values generated from each weeks prediction and actual values and demonstrates how these $R_{D}$ arrays overlap.

**Figure 8:**
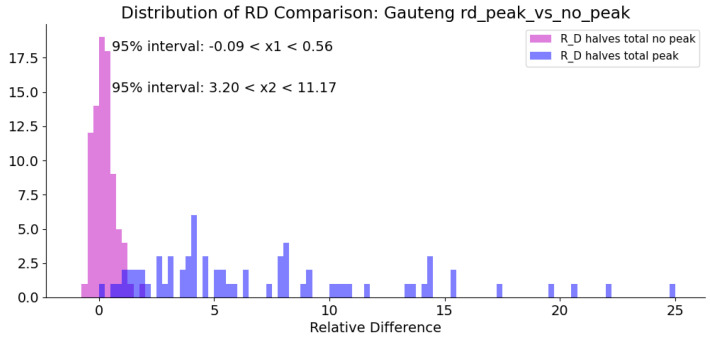
Distributions of $R_{D}$ during peak and non-peak periods.

Figure 8 shows the distribution of all of the first halves of the $R_{D}$ 14-day arrays created from each weeks prediction, labeled ‘1st 1/2s joined’ in Figure 7. The threshold value can then be extracted from this graph by choosing a value of $R_{D}$ that encloses the whole $R_{D}$ non-peak distribution. This is to take a conservative approach to issuing an alert. This is carried out for each province to identify the specific threshold values.

## 3. Results

### 3.1. Example Prediction Result during Non-Peak Period

**Figure 9:**
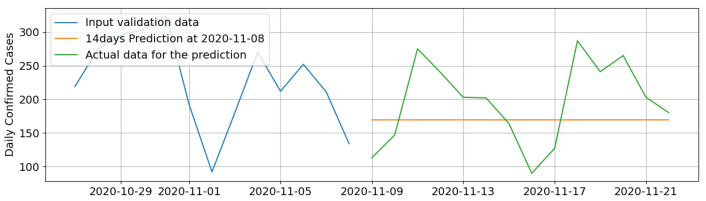
Graph showing 14-day prediction of dTCt during a non-peak period.

Figure 9 below shows the final hyper-parameter optimized LSTM RNN model’s ability to predict dTCt during non-peak times.

### 3.2. Verification of the Alert System Using Second Wave Data

Using the appropriate threshold discovered for each province in South Africa, shown in Table 4, the alert system was tested by comparing the system predicted start date of the second wave against the actual case data. The dates of the start of the second wave for each province obtained using this technique are shown in Table 5.

**Figure 10:**
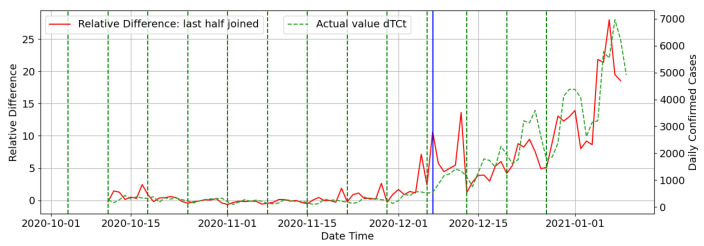
Graph showing all last halves of $R_{D}$ values joined: Gauteng.

Figure 10 below shows all the last halves of the $R_{D}$ values generated from each weeks prediction joined for a date period that extends into the second wave period for Gauteng province. The blue line indicates the date when the model identified a wave starting for Gauteng.

### 3.3. Third Wave Surveillance

**Figure 11:**
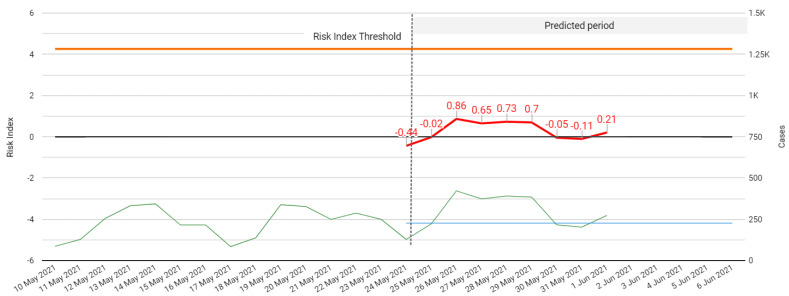
Graph showing the 14-day prediction of dTCt and rd values for surveillance of the 3rd wave.

Below is a screenshot of the final output of our model for third wave surveillance available on the COVID-19 monitoring website. (COVID-19 monitoring website: https://www.covid19sa.org/riskindex-ai (accessed on 29 June 2021)) The model shown on the site is updated weekly and the relative difference value is calculated automatically as new daily case data becomes available (Figure 11). Notably, at the time of writing, the system has been successful in detecting the beginning of the third wave in the provinces in South Africa. South African policy makers engage with the created RIM on a weekly basis during the Gauteng government COVID-19 command council meetings.

## 4. Limitations

The main limitations of this methodology relate to the availability of non-peak data for training data. If a province had a small period between the first and second peaks, the generated model might not be as good as some of the models developed for other provinces which have longer no-peak periods available for training.

## 5. Discussion and Conclusions

This research exploits the multivariate, multi-time-step time-series predictive capabilities of an RNN with LSTM to predict daily change in cases dTCt in South African provinces. Ten features were chosen as inputs to the RNN model. These features include mobility measures, stringency indicators and epidemiological parameters. The model was trained over the interim period between COVID-19 case waves within each province. This configuration caused the model to perform well over the interim period, however when another COVID-19 case wave is reached, the system is unable to predict the dTCt values accurately. The intentional pitfall of the model to predict dTCt during a peak has been taken advantage of to create an alert system by monitoring the relative difference $R_{D}$ between the prediction and the actual value on a daily basis. When the $R_{D}$ value is consistently above a calculated threshold for at least 2 days, the probability of an additional wave is high. The thresholds for each province are calculated by analysing the distributions of $R_{D}$ values generated as a result of the predictions over time during peak and non-peak times. The threshold was chosen by selecting an $R_{D}$ value that encapsulated the whole non-peak distribution.

Artificial Intelligence and Big Data can be exploited to devise complex, multi-dimensional, multi-variate, quantitatively reliable models that can assist public health decision- and policy-makers as well as physicians in a variety of tasks, including diagnosing COVID-19, identifying individuals at higher risk for COVID-19, stratifying patients and discovering potential treatments or verifying their effectiveness [22].

Recently, Artificial Intelligence and Big Data have also been utilized to predict COVID-19 relapses and resurgences [23]. Authors performed a comparative study, comparing countries such as the USA or Canada in which public health measures against COVID-19 had been implemented in a stringent way versus countries, such as Sweden, where policies were more relaxed, utilizing three different approaches (namely, a Bayesian susceptible-infected-recovered or SIR model, a Kalman filter, and machine learning). Policy interventions were effective in curbing the COVID-19 pandemic, even though the drop in infected cases was higher in those countries in which stricter policies had been enforced.

In the existing scholarly literature, there are few studies specifically utilizing RNN-based models aimed at predicting COVID-19 waves. For instance, Li and colleagues [24] have devised a RNN-based alert system, termed as Attentive Lockdown-awaRe Transfer Learning for Predicting COVID-19 Pandemics in Different Countries (ALeRT-COVID). This system was devised and trained on a pre-defined country (“source country”) and, then, adapted (“transferred”) to other target countries. Country-specific models have been implemented for Brazil [25], USA and India [26].

Few other studies have exploited mobility data, such as those generated by Google. For example, Wang et al. [27] have shown that is of paramount importance to understand dynamic changes in human mobility, social networks and spatial interaction trends to better predict the still ongoing COVID-19 pandemic. Authors were able to demonstrate that incorporating Google-outputted mobility data resulted in a significantly higher predictive power of COVID-19 cases.

In the present study, using our methodology, the dates of the starts of the second wave of COVID-19 cases in South African provinces were accurately estimated. Noteworthy, the dates generated by the model would not have been able to be achieved confidently by simply monitoring the daily change in cases only. Furthermore the model has been successful in identifying the start of the third wave of COVID-19 cases in South African provinces and has proved a valuable tool to South African policy makers.

## Figures and Tables

**Table 1:** Mobility Indicators.

Description	Indicators
Google Mobility	Retail and Recreation
	Grocery and Pharmacy
	Parks
	Transit Stations
	Workplaces
	Residential
Facebook Mobility	Tiles visited relative change
	Stay in place

**Table 2:** Hyper-parameters chosen for optimization and chosen possible values.

Hyper-Parameter	Value Options
Window size	[1, 3, 5, 7]
Number of LSTM layers	[1, 2, 3, 4]
Number of unites in LSTM layers	[5, 10, 15, 20]
Batch size	[5, 10, 15, 20]

**Table 3:** Methodology Comparison.

Prediction Method	RMSE
LSTM RNN model	76.57
Naive Forecast	89.43
Seasonal Naive Forecast	79.99

**Table 4:** Province Specific $R_{D}$ Threshold Values.

Province	$R_{D}$ Threshold
Gauteng	3.2
Western Cape	4.3
Eastern Cape	1.4
KwaZulu-Natal	13.4
Free State	0.8
Mpumalanga	2.0
Limpopo	3.0
Northern Cape	0.65
North West	1.3

**Table 5:** Province Specific Second Wave Start Date.

Province	2nd Second Wave Start Date
Gauteng	2020-12-07
Western Cape	2020-11-11
Eastern Cape	2020-10-21
KwaZulu-Natal	2020-12-01
Free State	2020-12-19
Mpumalanga	2020-12-15
Limpopo	2020-12-01
North West	2020-12-23
Northern Cape	2020-12-23

**Figure A1:**
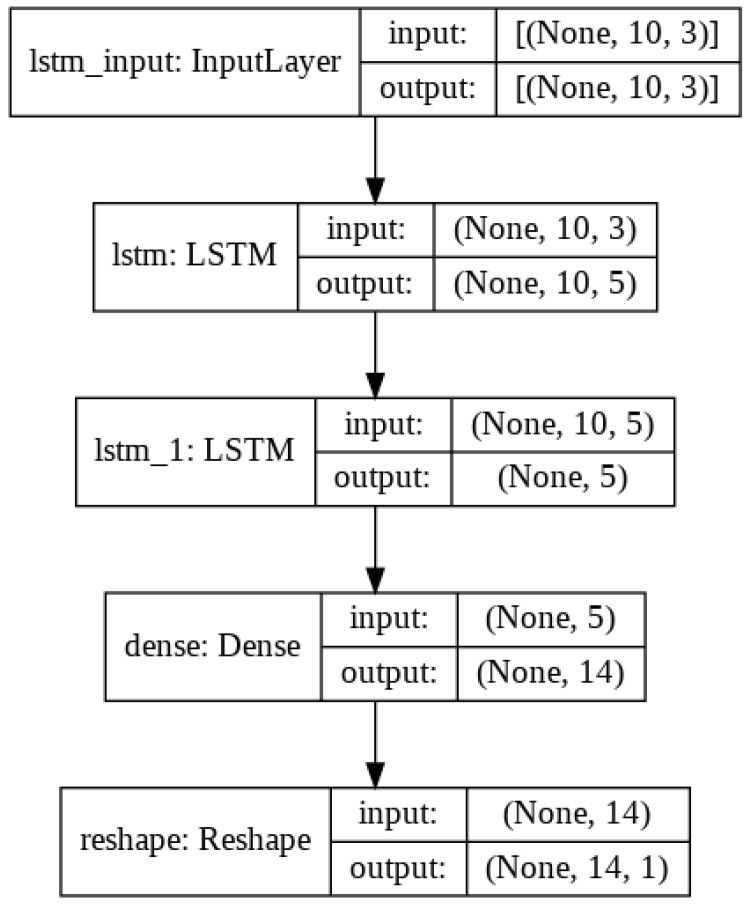
Schematic of Specific Neural Network Architecture.

## Data Availability

The data used in this study is publicly available at the following destinations: COVID-19 Case Data—https://www.nicd.ac.za/diseases-a-z-index/COVID-19/surveillance-reports/ (accessed on 29 June 2021), Facebook mobility data-set—https://data.humdata.org/dataset/movement-range-maps (accessed on 29 June 2021), Google mobility data-set—https://www.google.com/covid19/mobility/ (accessed on 29 June 2021), and OxCGRT stringency index—https://www.bsg.ox.ac.uk/research/research-projects/COVID-19-government-response-tracker (accessed on 29 June 2021).

## Appendix A. Neural Network Architecture

Figure A1 represents the specific neural network architecture used.
